# Supplementary material for: A Likelihood Approach for Real-Time Calibration of Stochastic Compartmental Epidemic Models
Source: PLoS Comput Biol. 2017 Jan 17;13(1):e1005257. doi: 10.1371/journal.pcbi.1005257 (PMC5240920; doi:10.1371/journal.pcbi.1005257)
Supplement: S1 File — (TAR.GZ) [file pcbi.1005257.s014.tar.gz › HSPH_Online-SI-Revision/PF08/n100-mild/n100-mild_PF2_summary2.pdf]

peak -8	3w spec	3w cum	5w spec	5w cum	end	3w spec LOG	3w cum LOG	5w spec LOG	5w cum LOG	end LOG
mean	138.5	86.1	79.2	79.4	75.5	0.4	0.3	0.5	0.4	0.3
median	55.4	46.8	67.8	60.5	71.9	0.4	0.3	0.6	0.4	0.2
quantile .1	17.7	17.9	23.9	18.2	49.8	0.1	0.1	0.1	0.1	0.2
quantile .9	217.2	192.6	150.	161.9	108.	0.7	0.6	0.9	0.1	0.5
31	36	27	30	11						
peak -4	3w spec	3w cum	5w spec	5w cum	end	3w spec LOG	3w cum LOG	5w spec LOG	5w cum LOG	end LOG
mean	24.5	21.	45.4	25.	115.7	0.1	0.1	0.2	0.1	0.3
median	20.	15.2	43.6	22.3	107.9	0.1	0.1	0.2	0.1	0.3
quantile .1	8.3	7.4	6.6	8.3	86.9	0.	0.	0.	0.	0.3
quantile .9	41.1	38.4	75.4	41.7	152.1	0.2	0.2	0.3	0.	0.4
17	24	9	20	0						
peak	3w spec	3w cum	5w spec	5w cum	end	3w spec LOG	3w cum LOG	5w spec LOG	5w cum LOG	end LOG
mean	193.	87.2	874.8	135.2	256.7	0.4	0.3	0.7	0.4	0.5
median	150.1	85.	348.2	126.	226.9	0.4	0.3	0.7	0.4	0.5
quantile .1	83.	42.8	160.3	71.3	170.4	0.3	0.2	0.4	0.2	0.4
quantile .9	268.1	140.6	880.9	203.1	356.1	0.6	0.4	1.	0.2	0.7
0	0	0	0	0						
peak +4	3w spec	3w cum	5w spec	5w cum	end	3w spec LOG	3w cum LOG	5w spec LOG	5w cum LOG	end LOG
mean	128 474.	1704.6	82 200.3	2271.4	3137.8	1.1	0.9	1.4	0.9	1.1
median	721.7	469.4	1499.3	575.4	875.7	0.9	0.8	1.2	0.8	1.
quantile .1	299.4	213.3	454.1	269.2	437.	0.6	0.5	0.7	0.6	0.7
quantile .9	2543.2	1242.2	9974.1	1745.6	2417.3	1.4	1.1	2.	0.6	1.4
0	0	0	0	0						
peak +8	3w spec	3w cum	5w spec	5w cum	end	3w spec LOG	3w cum LOG	5w spec LOG	5w cum LOG	end LOG
mean	200 120.	190 860.	280 845.	253 370.	334 949.	1.8	1.5	2.2	1.5	1.7
median	2822.1	1903.3	6150.	2249.4	2888.5	1.5	1.3	1.8	1.4	1.5
quantile .1	680.8	525.7	1261.3	631.	965.	0.9	0.8	1.1	0.9	1.
quantile .9	200 723.	14 998.4	1.02362×10^6	19 888.3	28 808.2	3.3	2.2	4.	0.9	2.5
0	0	0	0	0	{}	{}	{}	{}	{}	{}
